# No Compensatory Relationship between the Innate and Adaptive Immune System in Wild-Living European Badgers

**DOI:** 10.1371/journal.pone.0163773

**Published:** 2016-10-03

**Authors:** Yung Wa Sin, Chris Newman, Hannah L. Dugdale, Christina Buesching, Maria-Elena Mannarelli, Geetha Annavi, Terry Burke, David W. Macdonald

**Affiliations:** 1 Wildlife Conservation Research Unit, Department of Zoology, University of Oxford, Recanati-Kaplan Centre, Tubney House, Abingdon Road, Tubney, Abingdon, Oxfordshire, OX13 5QL, United Kingdom; 2 NERC Biomolecular Analysis Facility, Department of Animal and Plant Sciences, University of Sheffield, Sheffield, S10 2TN, United Kingdom; 3 Department of Organismic and Evolutionary Biology, Museum of Comparative Zoology, Harvard University, 26 Oxford Street, Cambridge, MA, 02138, United States of America; 4 Groningen Institute for Evolutionary Life Sciences, University of Groningen, PO Box 11103, 9700 CC, Groningen, Netherlands; 5 School of Biology, Faculty of Biological Sciences, University of Leeds, Leeds, LS2 9JT, United Kingdom; 6 School of Biological Sciences, University of East Anglia, Norwich Research Park, Norwich, Norfolk, NR4 7TJ, United Kingdom; 7 Faculty of Science, Department of Biology, University of Putra Malaysia, UPM 43400, Serdang, Selangor, Malaysia; Chang Gung University, TAIWAN

## Abstract

The innate immune system provides the primary vertebrate defence system against pathogen invasion, but it is energetically costly and can have immune pathological effects. A previous study in sticklebacks found that intermediate major histocompatibility complex (MHC) diversity correlated with a lower leukocyte coping capacity (LCC), compared to individuals with fewer, or many, MHC alleles. The organization of the MHC genes in mammals, however, differs to the highly duplicated MHC genes in sticklebacks by having far fewer loci. Using European badgers (*Meles meles*), we therefore investigated whether innate immune activity, estimated functionally as the ability of an individual’s leukocytes to produce a respiratory burst, was influenced by MHC diversity. We also investigated whether LCC was influenced by factors such as age-class, sex, body condition, season, year, neutrophil and lymphocyte counts, and intensity of infection with five different pathogens. We found that LCC was not associated with specific MHC haplotypes, MHC alleles, or MHC diversity, indicating that the innate immune system did not compensate for the adaptive immune system even when there were susceptible MHC alleles/haplotypes, or when the MHC diversity was low. We also identified a seasonal and annual variation of LCC. This temporal variation of innate immunity was potentially due to physiological trade-offs or temporal variation in pathogen infections. The innate immunity, estimated as LCC, does not compensate for MHC diversity suggests that the immune system may function differently between vertebrates with different MHC organizations, with implications for the evolution of immune systems in different taxa.

## Introduction

The innate and adaptive immune systems provide two lines of defence against pathogen invasion in vertebrates [1]. The innate immune system is activated quickly after pathogen challenge. Specific granular leukocytes, phagocytic neutrophils, are recruited immediately by chemical signals, such as chemokines, to the vicinity of infection. These leukocytes kill pathogens via oxidative mechanisms, termed the respiratory burst; a process in which neutrophils release reactive oxygen species (ROS), such as superoxides and hydrogen peroxides, to destroy invasive pathogens such as bacteria [2]. This ability of circulating neutrophils to produce this respiratory burst of ROS is known as the leukocyte coping capacity (LCC). Neutrophils also play a role in limiting replication of some virus strains [3]. In addition to killing small pathogens, neutrophils are able to sense pathogen size and kill large pathogens that are not readily phagocytosed [4]. The ROS produced by neutrophils may provide important protection against parasitic helminths [5, 6] and bites by ectoparasites [7]. Consequently, innate immunity plays a crucial role in immediate but non-specific defence against pathogens. In contrast, the adaptive immune system involves different leukocytes, known as lymphocytes, with antigen-specific functions and antigen-presenting cells, providing a highly specific immunological memory for pathogens, retained throughout the lifetime of an individual. These antigen-presenting cells, such as macrophages, B-cells and dendritic cells, express major histocompatibility complex (MHC) class II molecules on their surfaces, which bind and present pathogenic antigens to T-cells [8], in turn activating antibody production and other immune cascades. In addition, all nucleated somatic cells express MHC class I molecules that present antigens to cytotoxic T-cells [9].

MHC genes are the most polymorphic multi-gene family in the vertebrate genome [10]. For example, there are 447 and 271 alleles of HLA-B (class I) and DRB1 (class II) genes identified in humans [11]. The number of functional MHC loci varies significantly between different vertebrate [10] and mammalian species [12], evolving from birth-and-death processes, in which new genes arise by duplication and others are lost, or become non-functional. Furthermore, pathogen-mediated selective forces [13, 14] have been proposed to maintain the extreme diversity of MHC genes at the population level through balancing selection. Intra-individual MHC diversity, however, involves only a very small subset of this population diversity, according to the number of MHC loci per individual. Both the variation in the number of MHC loci and the heterozygosity at each locus contribute to the individual variability in the number of MHC alleles.

Each MHC molecule can only present peptides that match its antigen-binding sites. A higher number of different MHC molecules within an individual thus enables the presentation of a wider range of pathogenic antigens [15, 16]. Conversely, high intra-individual MHC diversity could result in a depletion of the mature T-cell repertoire [17]. This is because, during T-cell maturation in the thymus, a negative selection process eliminates T-cells with T-cell receptors (TCRs) that would otherwise react strongly with self-peptide−MHC complexes and cause autoimmune diseases [18]. The depletion of the TCR repertoire, due to high MHC diversity, also degrades immuno-competence. Consequently, intermediate MHC diversity, rather than maximum, is proposed to be optimal [19, 20].

To date, the three-spined stickleback, *Gasterosteus aculeatus* has been the principal subject of research into optimal intermediate MHC diversity and innate immunity. Given that they possess up to six MHC class IIB loci with a maximum of 12 alleles per individual [21], it is sticklebacks with an average of 5–6 different MHC alleles that harbor the lowest parasite intensities [22–24]. Interestingly, Kurtz *et al*. [24] found that intermediate MHC diversity in *G*. *aculeatus* also correlated with a lower respiratory burst reaction, compared to individuals with fewer or more MHC alleles. Because extended and/or strong expression of an innate immune response can be costly, due to the harmful side-effects of unquenched ROS [25], this research on sticklebacks implies a compensatory relationship between the innate immune system and an optimal adaptive immune system.

In contrast to the highly duplicated MHC class IIB genes in sticklebacks, MHC gene organization in mammals differs substantially [12]. The MHC class II gene region of mammals is subdivided into several gene clusters; for example, in humans, DR, DP, DM and DQ. Each cluster contains one or more functional β-chain gene(s) and a functional α-chain gene. The DRB gene is usually the most diverse among all class II MHC genes [26, 27], and in contrast to the DRB, the DRA, DQA and DQB have only one locus in many species [12]. The number of DRB loci in mammalian species typically ranges from one to three [12, 26, 28–30]. Since the MHC organization of mammals and sticklebacks is so different, here we investigate whether the compensatory relationship between the innate and adaptive immune systems reported in stickleback could also be identified in a mammal, the European badger (*Meles meles*; henceforth ‘badger’).

In badgers the DRB genes show a higher diversity compared to the DRA, DQA and DQB genes, which exhibit an almost uniform distribution of alleles among individuals [31]. Previous studies of the MHC in badgers indicated the presence of at least two class II DRB loci and two class I loci, with four and seven putatively functional sequences, respectively [31–33]. The number of two functional DRB loci in badgers are identical to closely related mammalian species (Bowen et al. 2006b; Weber et al. 2004), thus the badger provides an informative mammalian model to examine the influence of MHC diversity on innate immunity, especially given that Sin *et al*. (2014) have reported a MHC−pathogen association.

To estimate the respiratory burst reaction, as a functional estimate of innate immune activity [34], we measured Leukocyte Coping Capacity (LCC) [35]. Individuals with a higher LCC have a greater potential to produce a respiratory burst and are better able to respond to pathogen invasion [36, 37]. The ROS produced during the respiratory burst can, however, damage host tissue [25], creating a physiological trade-off. Here, we investigate whether LCC was influenced by age, sex, season, body condition and infection intensities with five different pathogens. We then investigate if an individual’s MHC diversity, or the presence/absence of specific MHC alleles or haplotypes, influences LCC. Finally, we determine if LCC is related to the number of two distinct white blood cell types: neutrophils and lymphocytes in the same blood samples.

## Materials and Methods

### Study population and sample collection

This study was conducted on a high-density badger population (36.4 ± 2.6 (SE) badgers/km^2^; [38]) in Wytham Woods (a 6 km^2^ deciduous woodland in Oxfordshire, UK; 51°46’26N, 1°19’19W). Detailed information of the population and sample collection are described in Macdonald *et al*. [38, 39] and Sin *et al*. [40]. Briefly, seasonal trapping events have been undertaken since 1987 [39], generally over two weeks in June (spring), September (summer), November (autumn), with occassional trapping in January (winter) [38]. Badgers were caught in mesh-traps baited with peanuts, placed near the entrances of active setts [38, 39]. All captured badgers were transported to a central handling facility and sedated by intra-muscular injection with ketamine hydrochloride [41]. Upon first capture all badgers were tattooed with a unique number on the left inguinal region for permanent individual identification. The sex, age-class (cub (<1 years old) or adult; [38]), weight (to the nearest 0.1 kg), body length (mm), and trapping location (social group affiliation) of each badger were recorded. Weight and body length were used to calculate a body condition index (weight/length ratio; [42]).

DNA samples were collected during sedation: ~100 guard hairs were plucked, and approximately 3 ml of blood was taken by jugular venipuncture using a vacutainer containing EDTA. Blood samples were aliquoted into sub-samples immediately for leukocyte coping capacity measurement and hematological analysis, or stored at -20°C for pathogen screening and MHC genotyping. Hair samples were preserved in 80% ethanol at room temperature until DNA isolation was performed. Faecal samples were also collected, for parasitological screening, by the administration of an enema consisting of 7.5 ml warm soapy water per kg bodyweight [43]. Faecal samples were preserved using 2.5% aqueous potassium dichromate (K_2_Cr_2_O_7_) at 4°C for later screening. Leukocyte coping capacity samples (n = 207 samples) were collected from individuals trapped in June, September and November of 2009 and 2010. Blood and faecal samples used for pathogen screening (n = 64) were collected from individuals trapped in June, September and November of 2009. Blood samples used for hematology analysis (n = 24) were collected from individuals trapped in November 2009. The blood and hair samples for MHC genotyping were collected across all study years from 1987 to 2010.

### Leukocyte coping capacity measurement

We used an in vitro challenge–coping approach to chemically stimulate a respiratory burst in whole blood [35, 44]. Ten microlitres of whole blood was transferred into a silicon anti-reflective tube (Lumivial, Berthold Technologies, Germany) containing 90 μl 10^−4^ mol l^-1^ luminol (5-amino-2,3-dihydrophthalzine; Sigma A8511) diluted in phosphate buffer (PBS; Sigma P4417). The tube was then shaken gently to mix the solution. This technique measures chemiluminescence produced in response to challenge triggered by adding 10 μl phorbol 12-myristate 13-acetate (PMA; Sigma P8139) at a concentration of 10^−3^ mol l^-1^. Dimethyl sulfoxide (DMSO; Sigma D5879) was first added to an amount that just dissolved the PMA completely, and then diluted to a final concentration of 10^−3^ mol l^-1^ in PBS. We used this PMA concentration because although trapping and transport stress may influence LCC [35], different concentrations of PMA (10^−3^, 10^−4^ and 10^−6^ mol l^-1^) tested on this species show that transport only had an effect on LCC in samples challenged with PMA at 10^−6^ but not 10^−3^ and 10^−4^ mol l^-1^[45]. Two replicates and one control tube, in which 10 μl PBS was added instead of PMA, were measured for each blood sample. Chemiluminescence was monitored every 5 min in a portable luminometer (Junior LB 9509, Berthold Technologies) over 90 min at 37°C. The area under curve (AUC), representing the overall oxygen radical production by neutrophils during these 90 min, was then calculated. The oxygen radical production of a sample was calculated as the average AUC of the two replicates subtracting the background from the control.

### Hematology

Hematological analysis was performed by the diagnostic laboratories of the Royal Veterinary College, University of London using a hematology analyser. These hematological results included white blood cell counts of neutrophils and lymphocytes. Neutrophils are important in innate immunity, while lymphocytes play a crucial role in adaptive immunity. The ratio of neutrophils to lymphocytes was also determined, to provide a rough estimation of the activity of the innate versus adaptive immune system.

### Pathogen screening

We examined a variety of pathogens including infection intensities of coccdia (*Eimeria melis*), trypanosome (*Trypanosoma pestanai*), mustelid herpes virus (MHV), as-well-as badger fleas (*Paraceras melis*) and badger lice (*Trichodectes melis*). Although 13 pathogens were determined in Sin *et al*. [40], only five species were measured consistently across the samples included in this study. Detailed screening methods are described in Sin *et al*. [40]. Briefly, a quantitative real-time PCR (qPCR) approach was used to determine the infection intensity of *T*. *pestanai* and MHV in the blood samples. The faecal flotation technique [46] was used to assess the intensity of *E*. *melis*. Badger fleas were counted during a 20 sec inspection of the badger’s body (for full details of this method see Cox et al. 1999). A standardized relative index of lice abundance was derived from inspection of a 4 x 4 cm square of skin in the inguinal region, prone to infestation (see [47]).

### MHC genotyping

Genomic DNA was isolated using the GFX Genomic Blood DNA Purification Kit (Amersham Biosciences, Little Chalfont, UK), following the scalable method in the manufacturer’s protocol, or from a minimum of 20 hairs with visible follicles, using a Chelex protocol [48]. The detailed method for MHC genotyping are described in Sin *et al*. [33]. Briefly, we used published primers to amplify exon 3 and exon 2 regions [31, 32] that encode the antigen-binding domain in MHC class I and class II DRB genes, respectively. These MHC sequences were separated by reference strand-mediated conformation analysis (RSCA), in which each ‘RSCA allele’ was confirmed to be a unique, putatively functional, sequence [49]. We used the number of alleles per individual as a measure of MHC heterozygosity across multiple loci [50, 51]. ‘Heterozygosity’ hereafter refers to the allelic diversity exhibited in class I and class II genes. MHC class II–class I haplotypes were included in the analysis and were calculated using parentage data by assuming Mendelian inheritance [33, 40]. Seven haplotypes were included in the analysis. The sampling size for haplotype analyses was smaller than that for MHC allele analyses, because haplotypes were inferred using parentage assignments provided by Annavi *et al*. [52], which limited the sample size. ‘Haplotype heterozygosity’ hereafter refers to the heterozygosity at the haplotype level.

### Data analyses

#### Multi-model inference

We employed linear mixed models to examine the influence of multiple factors by the inclusion of multiple explanatory variables and random effects [49, 53, 54]. Analyses were performed using the packages lme4 0.999375–42 [55], arm v1.8–6 [56], MuMIn v1.7.7 [57] and AICcmodavg v1.25 [58] in R 2.15.0 (R Core Development Team 2012). LCC, white blood cell densities and infection intensities of pathogens were log_10_ (intensity + 1) transformed, to correct for heterogeneity of variance. We used multi-model inference to establish which explanatory variables were influential, averaged over all plausible models [59–61]. Model selection was based on Akaike’s information criterion corrected for sample size (AICc; Akaike 1973). Models that are more plausible have lower AICc value. Multi-model inference [59] was performed for models with ΔAICc < 7 [62]. Model averaged parameter estimates and parameter estimates with shrinkage (i.e., parameter estimates set to zero in models that did not include the parameter) are reported. The unconditional standard errors and 95% confidence intervals [60] of parameter estimates are also reported, in order to allow model uncertainty to be included in both the model evaluation and the derivation of parameter estimates. The relative importance of a parameter was defined as the sum of Akaike weights (where the Akaike weight of each model is calculated as its relative likelihood (exp(-0.5*ΔAICc)) divided by the sum of Akaike weights of all models) for all models (ΔAICc < 7) including the predictor [59]. The parameter with the largest sum was inferred to be the most influential. The baseline sums of weights distribution for each predictor were calculated by performing 100 independent random permutations of the response in each dataset [63] to show when the predictors were not correlated to the response. The permutation tests showed baseline sums of weights have mean values range from 0−0.17, with most values smaller than 0.1 (Figs B−E in S1 Supplementary).

To determine the effect of MHC alleles and haplotypes, we first investigated whether LCC was related to different life-history factors by modeling five fixed effects (one continuous effect (body condition: weight/length ratio), four categorical effects (age-class: cub or adult; sex; season: spring, summer or autumn; year: 2009 or 2010)) and three interactions (season*year, sex*age, and sex*weight/length) and 2 random effects (individual and social group identities). Only significant fixed effects were retained in the second and third models to reduce the number of factors included in a single model. The second model (n = 171 samples; 122 badgers) included presence/absence of eight haplotypes (Sin et al. 2014) and the linear and quadratic effect of haplotype heterozygosity. In addition, we also undertook a third model (n = 207 samples; 153 badgers) using MHC class I and class II genes instead of haplotypes. The linear effect of heterozygosity of MHC class I and II genes, quadratic effect of heterozygosity of MHC class I genes, and presence/absence of five alleles (two class II DRB: Meme-DRB*01, and -DRB*04, Sin *et al*., 2012b; three class I: -MHCI*01, -MHCI*02, and -MHCI*04, Sin *et al*., 2012a) were included in these models. No quadratic effect of class II heterozygosity was included because individuals either possessed two or three alleles, i.e. only two levels identified. After accessing the variance inflation factors [64], Meme-DRB*03, -MHCI*03, and -MHCI*07 were not retained in the models because of high collinearity with MHC class II and I heterozygosity respectively. We controlled for individuals with multiple samples by including individual identity, and controlled for local effects by including social group identity as random effects in all models. All continuous predictors were standardized by mean centering and dividing by two standard deviations using the R package arm [65] to allow direct comparison of sizes of effects across different scales [66, 67].

Since pathogen infection intensities were only determined for 2009 samples (n = 64 samples), we tested whether pathogen infection affected the leukocyte coping capacity in a separate model. We included infection intensities of the five pathogens (*E*. *melis*, *T*. *pestanai*, MHV, *P*. *melis* and *T*. *melis*) as fixed effects in the model, together with those factors in model one, excluding the year effect terms. Individual and social group identities were included as random effects.

We also investigated whether hematological parameters (n = 24 samples, from 24 individuals) were related to leukocyte coping capacity by including neutrophils count, lymphocytes counts, and neutrophils/lymphocytes ratio (N/L ratio) as fixed effects in a separate model, together with weight/length ratio, age-class, and sex. Social group identity was included as random effect.

## Results

### LCC

Over the 90 min of measurement, stimulus-induced oxygen radical production reached its highest value quickly (50% of samples peaked before 15 min, and 87% before 30 min) and then slowly returned to baseline.

### LCC and MHC genes

**Fig 1:**
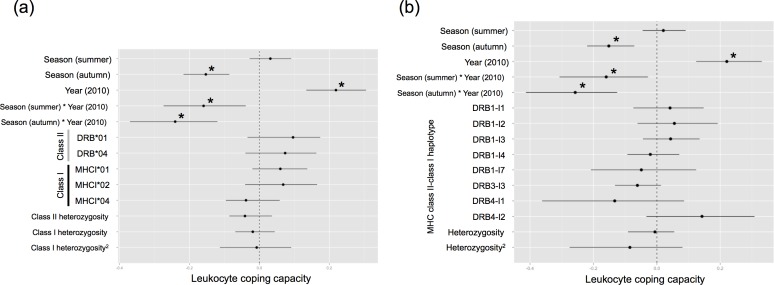
Model averaged parameter estimates and their 95% confidence intervals for the 3 predictors (season, year, season*year), and (a) presence/absence of three MHC class I and two MHC class II genes, linear effect of class II heterozygosity, and linear and quadratic effects of class I heterozygosity, or (b) presence/absence of MHC class II−class I haplotypes, and linear and quadratic effects of haplotype heterozygosity associated with the leukocyte coping capacity. * indicates a parameter with a significant effect. Spring and year 2009 were the reference categories.

In the first model, which included life-history factors, no association between LCC and age-class, sex, weight/length ratio, age*sex, and sex*weight/length was found (Table A in S1 Supplementary). Significant fixed effects involving season and year were included in the second and third models. There was no significant association between LCC and the presence of specific MHC alleles and haplotypes (Fig 1; Tables B and C in S1 Supplementary). There was no association between LCC and linear or quadratic effects of MHC heterozygosity at class I genes, class II genes, or class II–class I haplotypes (Fig 1).

There was an interaction between seasonal and annual differences in LCC (Fig 1; Tables B and C in S1 Supplementary). LCC was higher in summer than in spring in 2009 but lower in summer than in spring in 2010 (Fig 1; Tables B and C in S1 Supplementary; Fig A in S1 Supplementary). The difference between LCC in autumn compared to spring was higher in 2010 than 2009, where LCC was much lower in autumn than in spring in 2010 than in 2009 (Fig 1; Tables B and C in S1 Supplementary; Fig A in S1 Supplementary).

### Innate immunity and pathogen intensity

**Fig 2:**
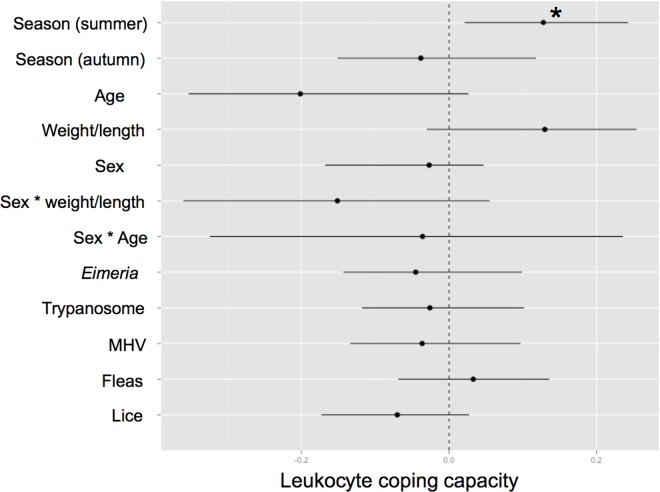
Model averaged parameter estimates and their 95% confidence intervals for the predictors (season, age, weight/length ratio, sex, sex*weight/length, sex*age, and infection intensity of five pathogens) associated with the leukocyte coping capacity. * indicates a parameter with a significant effect.

There was no association between LCC and infection intensities with the five pathogens (*E*. *melis*, *T*. *pestanai*, MHV, *P*. *melis* and *T*. *melis*) examined in 2009 (Fig 2; Table D in S1 Supplementary). Seasonal variation was also detected, where summer samples exhibited a higher LCC than spring samples (Fig 2; Table D in S1 Supplementary). There was no association between LCC and age-class, sex, weight/length ratio, age*sex, and sex*weight/length (Table D in S1 Supplementary).

### Innate immunity and white blood cell counts

**Fig 3:**
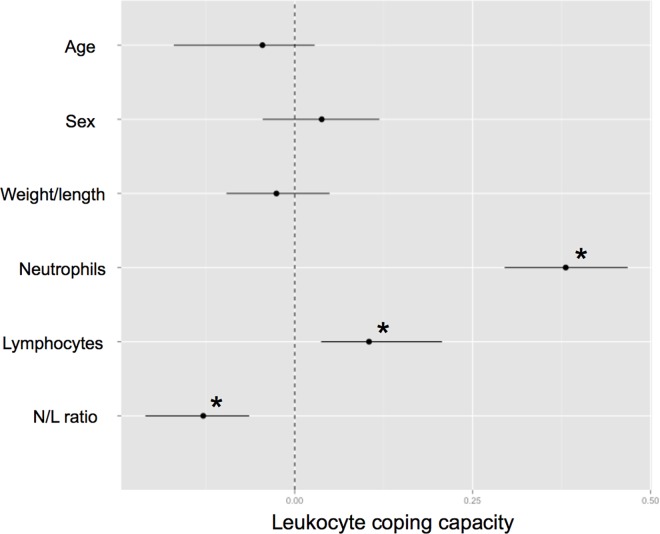
Model averaged parameter estimates and their 95% confidence intervals for the 6 predictors (age class, sex, weight/length ratio, neutrophil counts, lymphocyte counts and neutrophil/lymphocyte ratio) associated with the leukocyte coping capacity. * indicates a parameter with a significant effect.

There was a positive association between LCC and both neutrophil and lymphocyte counts (Fig 3; Table E in S1 Supplementary). The N/L ratio had a negative association with LCC, which means a higher LCC correlated with a greater increase in the number of lymphocytes than neutrophils. No association between LCC and age-class, sex, and weight/length ratio was found (Table E in S1 Supplementary).

## Discussion

In contrast to the complementary way that the innate and adaptive immune systems interact in the stickleback, where the lowest respiratory bursts were produced by individuals with optimal intermediate allelic diversity [24], we found no association between LCC and MHC heterozygosity in these European badgers. This difference between the two species likely may be due to the difference in their MHC organization. Sticklebacks possess up to six MHC class IIB loci [21], which can theoretically comprise 1–12 different MHC alleles. The effect of extreme MHC diversity would therefore be more prominent in sticklebacks than in badgers, i.e. sticklebacks with extremely low MHC diversity have a much lower MHC repertoire for antigen presentation, and sticklebacks with extremely high MHC diversity have a much more depleted TCR repertoire [17], compared to badgers. Since European badgers only have two MHC class II DRB loci (and probably two class I loci), and one of these is monomorphic [31–33, 40], the difference in MHC and TCR repertoires between individuals with different MHC diversity will be much smaller compared to sticklebacks. In addition, badgers do not appear to exhibit a general MHC heterozygote advantage with regard to pathogen resistance, based on evidence by Sin *et al*. (2014), which showed that the MHC heterozygote advantage against pathogens was much less common compared to the MHC allele-pathogen association. Consequently, in the badger, the innate immune system does not need to function in a complementary way to the adaptive immune system, with respect to extremely high or low MHC diversity. Alternatively, badgers and sticklebacks may have different susceptibility to oxidative damage arising from unquenched ROS, due to differences in basal metabolic rates between endotherms and ectotherms [68] or lifespan [69], leading to the difference in the extent they can use LCC to compensate MHC diversity.

There was no association between LCC and specific MHC alleles/haplotypes. Although individuals that possessed particular alleles/haplotypes had greater susceptibility to particular pathogen(s) compared to those without these alleles/haplotypes [40]; no susceptible alleles/haplotypes were associated with higher LCC. There was also no association between LCC and the infection intensity of pathogens examined in this study. Since association between MHC genes and pathogen resistance, or susceptibility, is apparent in badgers [40], the lack of association between LCC and MHC allele/haplotype or pathogen intensity indicates that even though the adaptive immune system might not be able to resolve infection efficiently, the innate immune system was not acting in a compensatory way such as seen in the sticklebacks.

There are just a handful of studies investigating the associations between MHC heterozygosity and immune responsiveness *per se*. Except for the study of Kurtz et al. [24] that used LCC as an estimate of innate immune response, other studies estimated immune responsiveness using techniques such as the phytohaemagglutinin (PHA) assay [70–72]. The PHA assay uses injected PHA to stimulate localized inflammation, which reflects the ability of an organism to mount a cell-mediated immune response [73]. By quantifying the swelling response of the skin, the immuno-competence of both the innate and adaptive immune systems is estimated. Studies on humans, house sparrows and water voles have identified association between response to PHA and MHC alleles, but not MHC diversity [70–72]. Since PHA triggers both innate and adaptive immune responses, the use of estimates of innate immune response could give different results. In fact, we also identified an association between LCC and particular MHC alleles and haplotype (data not shown), but the effect disappeared after we included social group identity as a random factor in our models, as those alleles/haplotype occurred primarily within a single social group.

Neutrophils produce ROS upon activation, and we show that the number of circulating neutrophils was the major factor driving LCC in badgers, i.e., a greater LCC correlated positively with an increased neutrophil count. In addition to neutrophils, lymphocyte counts also correlated positively with LCC, which suggests that current infection was triggering both the innate and adaptive immune responses. The negative relationship between LCC and N/L ratio, which indicates the relative activities of the innate and adaptive immune systems in terms of white blood cell production, showed that the immune system was activated to produce more lymphocytes than neutrophils during a potential infection when LCC was high. Elevated levels of both neutrophils and lymphocytes support that the innate and adaptive immune systems are inter-dependent parts of a single integrated immune system [74]. The innate immune response provides a signal to mount an adaptive immune response, and the adaptive immune response calls on the innate immune system to kill invading pathogens [74]. Moreover, neutrophils have been reported to express MHC class I and class II molecules and are able to influence adaptive responses by presenting antigens to induce T-cell proliferation [75, 76].

We also demonstrate seasonal variation in badger immunity. Seasonal variation in LCC could be due to physiological trade-offs, by which organisms regulate allocation of limited resources to multiple energetically costly functions [77, 78]. A key trade-off has been proposed to involve the reproductive and immune systems, where physiological changes that happen during reproduction (e.g. hormonal changes) may influence the immune system of an organism [77, 79]. Fluctuations in immune response occur in a variety of taxa [80], and in some cases seasonal variation in immunity are concurrent with breeding season (e.g. [81]). Sex hormones, such as testosterone, involves in trade-offs between the immune and reproductive systems [82, 83]. Nevertheless, the immuno-suppressive effects of testosterone are not consistent across taxa [84, 85]. The European badger has a polygynandrous mating system [86] and can mate throughout the year, but has mating peaks in late winter and late summer [87, 88]. Male badgers exhibit testosterone peaks during these mating seasons, but then the testes ascend and testosterone levels decrease in the autumn [87]. A low LCC in autumn was apparent in both years, which does not fit the prediction of an immuno-suppressive effect of testosterone. Importantly, male and female badgers, which have different testosterone levels [87], were not different in their LCC. Another possibility is that, given that resource limitation determines investment in immunity, individuals in better condition should be able to mount more effective immune responses than those in poor condition [89]. Body condition, measured as weight/length ratio, did not however influence LCC.

The pattern of variation in LCC was different between the two years we examined, and there were annual variations in seasonal effects. An alternative hypothesis as to why LCC varied with the season and year involves changes in pathogen abundance over time, as seen for many badger pathogens [40, 90]. Temporal variation in immune responses may therefore indicate an effort to fight off infection at those times of the year when infection risk is the highest. Future study of innate immune response in badgers should include measures of the prevalence and intensity of pathogen infections, for pathogens that show a seasonal variation.

Badger cubs generally have a higher pathogen load than do adults (see [40, 43]), and since cubs are more likely than adults to be encountering infections for the first time, they are more likely to mount a higher innate immune response. Age-class did not, however, influence LCC. Interestingly, badger cubs exhibit higher non-enzymatic plasma antioxidant capacity, expressed as vitamin E analogue, than adults aged six years and over [91]. The ROS produced during respiratory burst can cause oxidative damages leading to cell deaths, and this immuno-pathological effect is mitigated by antioxidant defences [92]. Therefore, cubs appear to have a higher antioxidant level than adults in order to mitigate the stronger (or more frequent) oxidative stress produced by LCC. Further research is needed to clarify the role of the innate and adaptive immune systems at different life stages in this species.

Understanding immune system regulation at different life stages is important for studying the relationship between the immune system and reproductive investment. Trade-off between antioxidant investment in immuno-competence versus developing and maintaining secondary sexual traits has been reported, especially for carotenoid-based visual traits [92], e.g. the red ornamentation of male sticklebacks [93]. In contrast, badgers rely more on olfactory signals than visual signals for communication, with individual-specific odor generated by subcaudal glands [94], where secretion volume is correlated positively with body condition and male reproductive status [95]. Interestingly, vitamin E has been found in the subcaudal gland secretion (unpublished data), which could be a secondary sexual trait to advertise antioxidant defence ability, or could be just a by-product from the activities of a diverse microbial community [96]. Many other mammals use scent glands as secondary sexual traits, for example the flank glands of male water voles function as an indicator of their social status and possibly genetic qualities [97]. The association identified between the water vole flank gland size and response to PHA [71] suggests that immuno-competence and development of scent glands as secondary sexual traits could also be a trade-off in badgers and other mammals.

In conclusion, we revealed that innate immunity, indicated by LCC, was not associated with specific MHC alleles/haplotypes and MHC heterozygosity in a mammal species. This indicates that the innate immune system does not compensate for any deficiencies arising from susceptible MHC alleles. This could be due to the high energetic trade-off costs of mounting an innate immune response and/or due to its immuno-pathological effects [78]. We discovered both seasonal and annual variations of LCC, which could be due to a physiological trade-off or temporal variation of pathogens. We show that it is crucial to establish how the MHC genes, oxidative stress and antioxidant defences interact with each other; where understanding how different species resist and respond to disease, and the trade-offs involved, is critical for informing conservation management.

## Supporting Information

S1 Supplementary Table A The association of leukocyte coping capacity in badgers with life-history factors; Table B The association of leukocyte coping capacity in badgers with presence or absence of alleles and MHC heterozygosity; Table C The association of leukocyte coping capacity in badgers with presence or absence of MHC class II-class I haplotypes and haplotype heterozygosity; Table D The association of leukocyte coping capacity in badgers with pathogen intensities in 2009; Table E The association of leukocyte coping capacity in badgers with white blood cell counts and ratio; Fig A Plots of leukocyte coping capacity against season (Spring, Summer and Autumn), for the years 2009 and 2010; Fig B Baseline sum of weights for each predictor from 100 permutations of the response variable for model 3 (MHC alleles); Fig C Baseline sum of weights for each predictor from 100 permutations of the response variable for model 2 (MHC haplotypes); Fig D Baseline sum of weights for each predictor from 100 permutations of the response variable for LCC and pathogen model; Fig E Baseline sum of weights for each predictor from 100 permutations of the response variable for LCC and white blood cell counts model.
(PDF)

S2 Supplementary LCC and MHC alleles data. (TXT)

S3 Supplementary LCC and MHC haplotypes data. (TXT)

S4 Supplementary LCC and pathogen data. (TXT)

S5 Supplementary LCC and hematology data. (TXT)
