# Supplementary material for: No Compensatory Relationship between the Innate and Adaptive Immune System in Wild-Living European Badgers
Source: PLoS One. 2016 Oct 3;11(10):e0163773. doi: 10.1371/journal.pone.0163773 (PMC5047587; doi:10.1371/journal.pone.0163773)
Supplement: S1 Supplementary — Table A The association of leukocyte coping capacity in badgers with life-history factors; Table B The association of leukocyte coping capacity in badgers with presence or absence of alleles and MHC heterozygosity; Table C The association of leukocyte coping capacity in badgers with presence or absence of MHC class II-class I haplotypes and haplotype heterozygosity; Table D The association of leukocyte coping capacity in badgers with pathogen intensities in 2009; Table E The association of leukocyte coping capacity in badgers with white blood cell counts and ratio; Fig A Plots of leukocyte coping capacity against season (Spring, Summer and Autumn), for the years 2009 and 2010; Fig B Baseline sum of weights for each predictor from 100 permutations of the response variable for model 3 (MHC alleles); Fig C Baseline sum of weights for each predictor from 100 permutations of the response variable for model 2 (MHC haplotypes); Fig D Baseline sum of weights for each predictor from 100 permutations of the response variable for LCC and pathogen model; Fig E Baseline sum of weights for each predictor from 100 permutations of the response variable for LCC and white blood cell counts model. (PDF) [file pone.0163773.s001.pdf]

S1 Supplementary materials

Table A The association of leukocyte coping capacity in badgers with life-history factors (for model 3 (MHC alleles), number of samples = 207; number of badgers = 153; for model 2 (MHC haplotypes), number of samples = 171; number of badgers = 122). Parameter estimates are provided with their unconditional standard errors (SE), 95% confidence intervals and relative importance, after model averaging (models with ΔAICc < 7). Estimates where 95% confidence intervals do not overlap zero are highlighted in bold.

Parameter	Estimate	Unconditional SE	95% Confidence interval		Relative importance^
For model 3 (MHC alleles) [a]					
(Intercept)	**6.339**	**0.030**	**6.279**	**6.395**	
Season (summer)	0.029	0.030	-0.028	0.091	1.00
Season (autumn)	**-0.156**	**0.033**	**-0.217**	**-0.087**	**1.00**
Year (2010)	**0.220**	**0.044**	**0.134**	**0.305**	**1.00**
Season (summer)*Year (2010)	**-0.157**	**0.060**	**-0.273**	**-0.038**	**1.00**
Season (autumn)*Year (2010)	**-0.247**	**0.064**	**-0.370**	**-0.120**	**1.00**
Weight/length	0.027	0.029	-0.033	0.081	0.38
Sex	0.014	0.028	-0.042	0.068	0.35
Age	-0.003	0.035	-0.061	0.077	0.27
Sex*weight/length	-0.081	0.054	-0.187	0.026	0.08
Age*sex	-0.043	0.067	-0.175	0.088	0.02
For model 2 (MHC haplotypes) [b]					
(Intercept)	**6.326**	**0.033**	**6.261**	**6.390**	
Season (summer)	0.023	0.035	-0.043	0.092	1.00
Season (autumn)	**-0.145**	**0.038**	**-0.217**	**-0.069**	**1.00**
Year (2010)	**0.231**	**0.052**	**0.128**	**0.334**	**1.00**
Season (summer)*Year (2010)	**-0.172**	**0.071**	**-0.310**	**-0.032**	**1.00**
Season (autumn)*Year (2010)	**-0.274**	**0.073**	**-0.417**	**-0.131**	**1.00**
Weight/length	0.019	0.034	-0.055	0.078	0.32
Sex	0.003	0.033	-0.062	0.067	0.31
Age	-0.035	0.041	-0.105	0.057	0.31
Sex*weight/length	-0.099	0.062	-0.221	0.024	0.07
Age*sex	-0.045	0.078	-0.197	0.108	0.02

^ Relative importance describes the sum of Akaike weights for all models (ΔAICc < 7) including the parameter

[a] Variances of random effects: individual identity: 0.0081 (S.D. = 0.090); social group identity: 0.0080 (S.D. = 0.088); residual: 0.023 (S.D. = 0.15)

[b] Variances of random effects: individual identity: 0.0095 (S.D. = 0.097); social group identity: 0.0077 (S.D. = 0.088); residual: 0.024 (S.D. = 0.15)

Table B The association of leukocyte coping capacity in badgers (number of samples = 207; number of badgers = 153) with presence or absence of alleles and MHC heterozygosity. Parameter estimates are provided with their unconditional standard errors (SE), 95% confidence intervals and relative importance, after model averaging (models with ΔAICc < 7). Estimates where 95% confidence intervals do not overlap zero are highlighted in bold.

Parameter	Estimate	Unconditional SE	95% Confidence interval		Relative importance^
(Intercept)	**6.337**	**0.030**	**6.278**	**6.395**	
Season (summer)	0.031	0.030	-0.028	0.091	1.00
Season (autumn)	**-0.153**	**0.033**	**-0.217**	**-0.086**	**1.00**
Year (2010)	**0.219**	**0.044**	**0.134**	**0.305**	**1.00**
Season (summer)*Year (2010)	**-0.160**	**0.060**	**-0.274**	**-0.039**	**1.00**
Season (autumn)*Year (2010)	**-0.241**	**0.064**	**-0.370**	**-0.120**	**1.00**
DRB*01 (present)	0.096	0.053	-0.034	0.174	0.56
DRB*04 (present)	0.074	0.052	-0.040	0.163	0.43
MHCI*01 (present)	0.060	0.040	-0.020	0.137	0.47
MHCI*02 (present)	0.068	0.053	-0.041	0.165	0.40
MHCI*04 (present)	-0.038	0.039	-0.096	0.058	0.33
Class II heterozygosity	-0.041	0.031	-0.086	0.036	0.40
Class I heterozygosity	-0.019	0.029	-0.069	0.044	0.28
Class I heterozygosity^2	-0.008	0.052	-0.113	0.091	0.25

^ Relative importance describes the sum of Akaike weights for all models (ΔAICc < 7) including the parameter

Variances of random effects: individual identity: 0.0062 (S.D. = 0.079); social group identity: 0.0075 (S.D. = 0.087); residual: 0.025 (S.D. = 0.15)

Table C The association of leukocyte coping capacity in badgers (number of samples = 171; number of badgers = 122) with presence or absence of MHC class II-class I haplotypes and haplotype heterozygosity. Parameter estimates are provided with their unconditional standard errors (SE), 95% confidence intervals and relative importance, after model averaging (models with ΔAICc < 7). Estimates where 95% confidence intervals do not overlap zero are highlighted in bold.

Parameter	Estimate	Unconditional SE	95% Confidence interval		Relative importance^
(Intercept)	**6.337**	**0.035**	**6.261**	**6.398**	
Season (summer)	0.020	0.035	-0.045	0.091	1.00
Season (autumn)	**-0.151**	**0.038**	**-0.220**	**-0.071**	**1.00**
Year (2010)	**0.221**	**0.053**	**0.124**	**0.331**	**1.00**
Season (summer)*Year (2010)	**-0.160**	**0.071**	**-0.307**	**-0.028**	**1.00**
Season (autumn)*Year (2010)	**-0.257**	**0.073**	**-0.413**	**-0.125**	**1.00**
DRB1-I3	0.044	0.046	-0.044	0.135	0.26
DRB1-I7	-0.049	0.085	-0.208	0.124	0.21
DRB1-I1	0.042	0.056	-0.074	0.148	0.24
DRB1-I2	0.055	0.064	-0.061	0.192	0.25
DRB3-I3	-0.061	0.037	-0.132	0.013	0.48
DRB4-I1	-0.133	0.114	-0.362	0.086	0.35
DRB4-I2	0.142	0.087	-0.033	0.308	0.55
DRB1-I4	-0.021	0.042	-0.093	0.071	0.21
Haplotype heterozygosity	-0.006	0.037	-0.091	0.055	0.22
Haplotype heterozygosity^2	-0.085	0.091	-0.275	0.081	0.28

^ Relative importance describes the sum of Akaike weights for all models (ΔAICc < 7) including the parameter

Variances of random effects: individual identity: 0.0076 (S.D. = 0.087); social group identity: 0.0081 (S.D. = 0.090); residual: 0.025 (S.D. = 0.16)

Table D The association of leukocyte coping capacity in badgers (n = 64 samples; n = 57 badgers) with pathogen intensities in 2009. Parameter estimates are provided with their unconditional standard errors (SE), 95% confidence intervals and relative importance, after model averaging (models with ΔAICc < 7). Estimates where 95% confidence intervals do not overlap zero are highlighted in bold.

Parameter	Estimate	Unconditional SE	95% Confidence interval		Relative importance^
(Intercept)	**6.210**	**0.046**	**6.165**	**6.343**	
Season (summer)	**0.128**	**0.056**	**0.021**	**0.243**	**0.98**
Season (autumn)	-0.038	0.068	-0.151	0.118	0.98
Age	-0.202	0.097	-0.353	0.026	0.73
Weight/length	0.130	0.073	-0.030	0.254	0.60
Sex	-0.027	0.055	-0.168	0.047	0.25
Sex*weight/length	-0.151	0.106	-0.360	0.055	0.06
Age*Sex	-0.036	0.143	-0.324	0.236	0.02
Eimeria	-0.045	0.062	-0.143	0.099	0.21
Trypanosome	-0.026	0.056	-0.118	0.102	0.16
MHV	-0.036	0.059	-0.134	0.097	0.17
Fleas	0.033	0.052	-0.069	0.136	0.18
Lice	-0.070	0.051	-0.173	0.027	0.35

^ Relative importance describes the sum of Akaike weights for all models (ΔAICc < 7) including the parameter

Variances of random effects: individual identity: 0.020 (S.D. = 0.14); social group identity: 0.0081 (S.D. = 0.090); residual: 0.014 (S.D. = 0.12)

Table E The association of leukocyte coping capacity in badgers (n = 24 samples; n = 24 badgers) with white blood cell counts and ratio. Parameter estimates are provided with their unconditional standard errors (SE), 95% confidence intervals and relative importance, after model averaging (models with ΔAICc < 7). Estimates where 95% confidence intervals do not overlap zero are highlighted in bold.

Parameter	Estimate	Unconditional SE	95% Confidence interval		Relative importance^
(Intercept)	**6.225**	**0.018**	**6.189**	**6.259**	
Age	-0.045	0.050	-0.170	0.028	0.19
Weight/length	-0.026	0.037	-0.096	0.049	0.15
Sex	0.038	0.042	-0.045	0.119	0.21
Neutrophils	**0.381**	**0.044**	**0.295**	**0.468**	**1.00**
Lymphocytes	**0.104**	**0.043**	**0.037**	**0.207**	**0.42**
Neutrophil/lymphocyte ratio	**-0.129**	**0.037**	**-0.210**	**-0.064**	**0.64**

^ Relative importance describes the sum of Akaike weights for all models (ΔAICc < 7) including the parameter

Variances of random effects: social group identity: 0.0 (S.D. = 0.0); residual: 0.0066 (S.D. = 0.081)

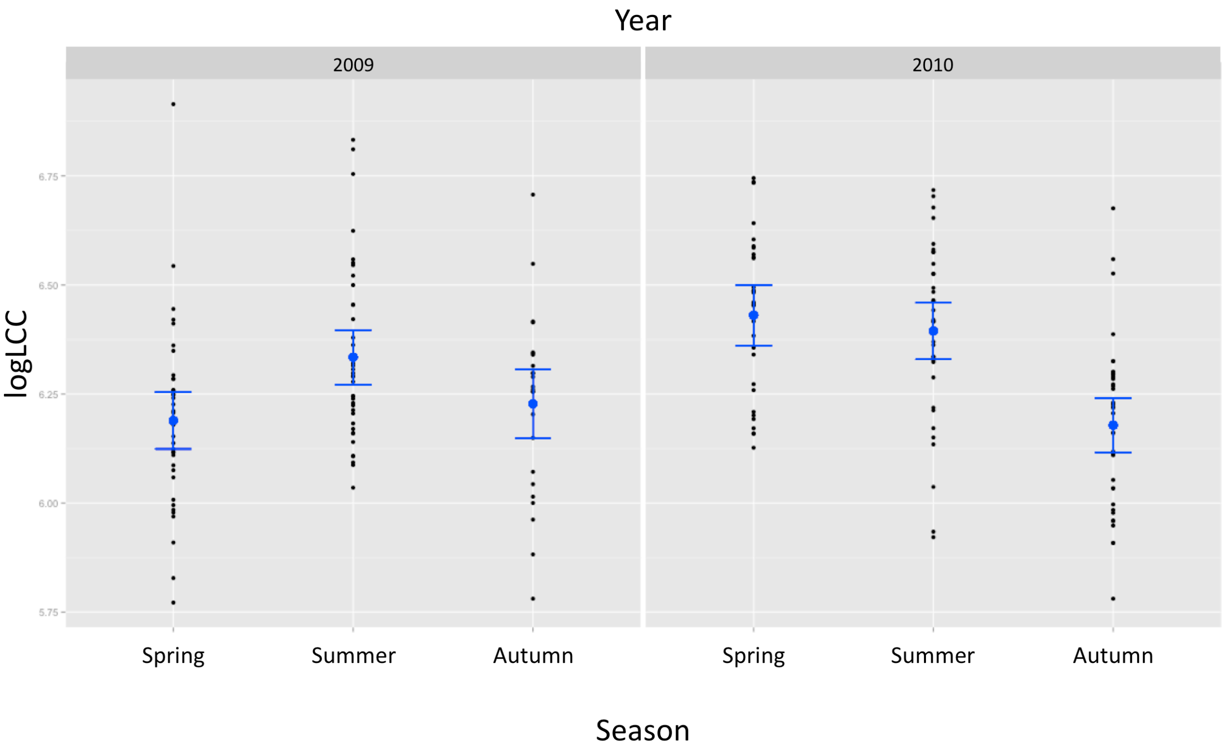

Figure A Plots of leukocyte coping capacity against season (Spring, Summer and Autumn), for the years 2009 and 2010. Black dot indicates the raw data. Blue dot indicates the predicted mean ± CI, which was calculated using the function *predict* in the R package *ggplot2* from the linear model logLCC ~ Season*Year.

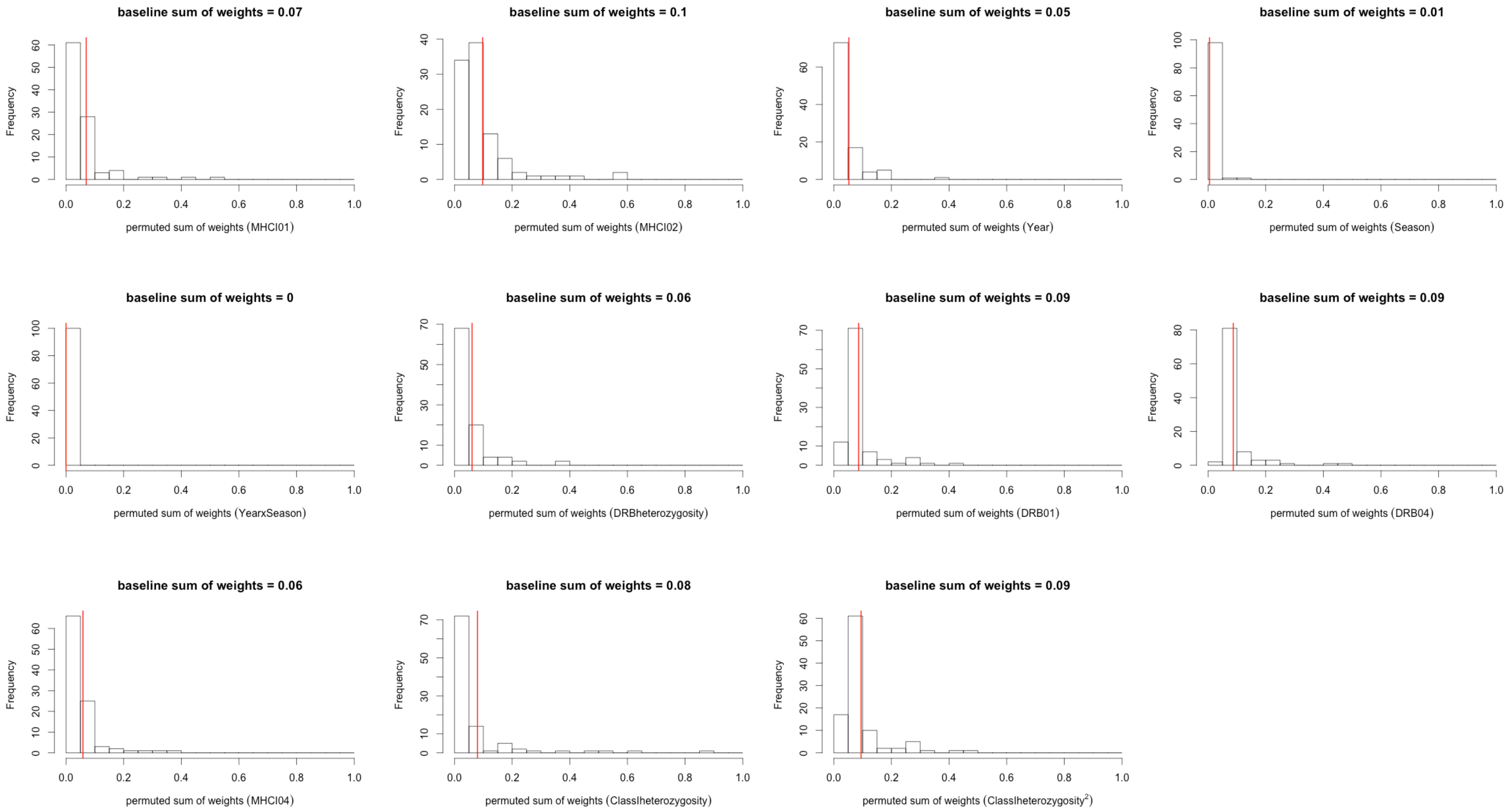

Fig. B Baseline sum of weights for each predictor from 100 permutations of the response variable for model 3 (MHC alleles). Mean values are indicated with the red line.

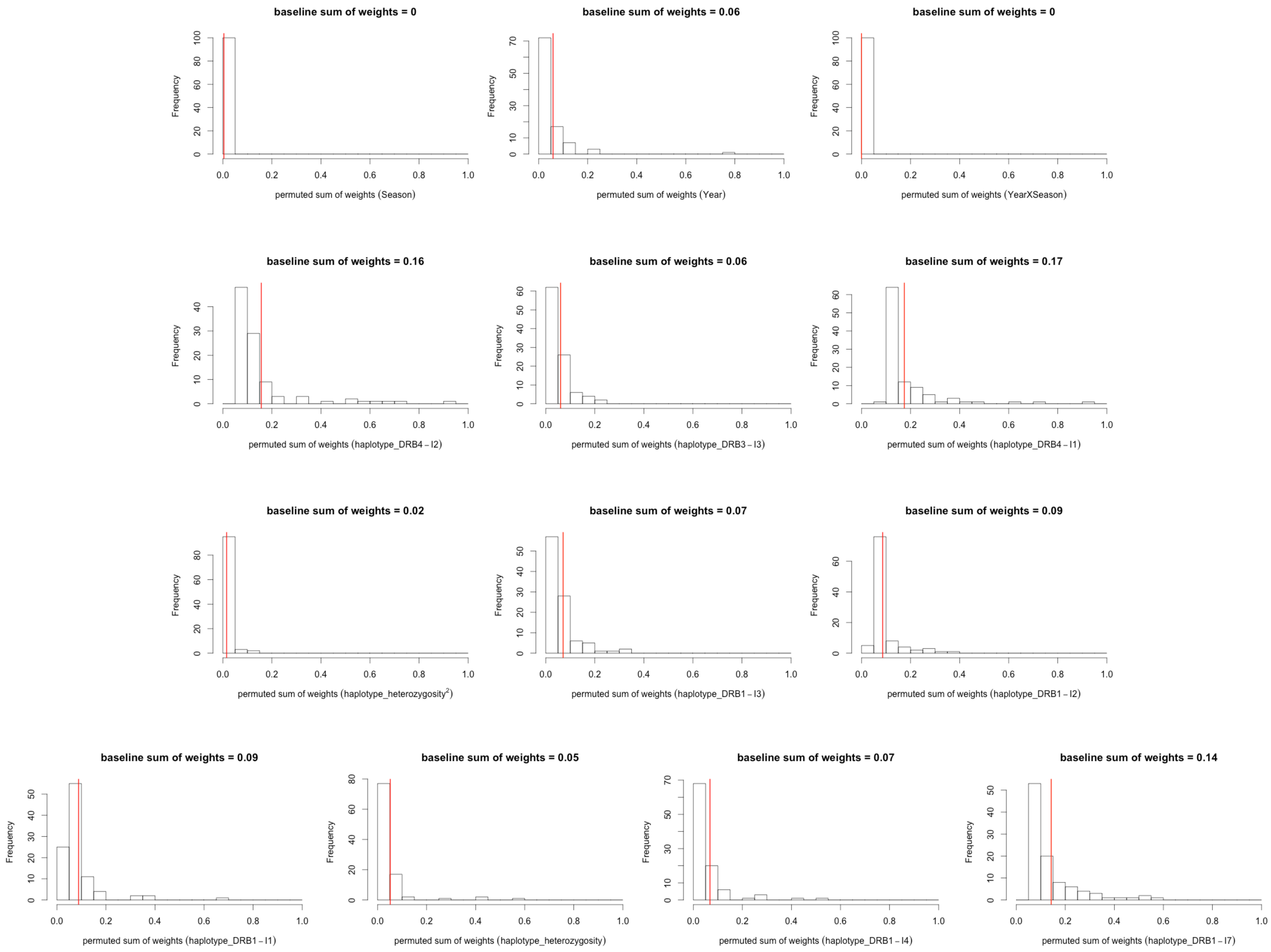

Fig. C Baseline sum of weights for each predictor from 100 permutations of the response variable for model 2 (MHC haplotypes). Mean values are indicated with the red line.

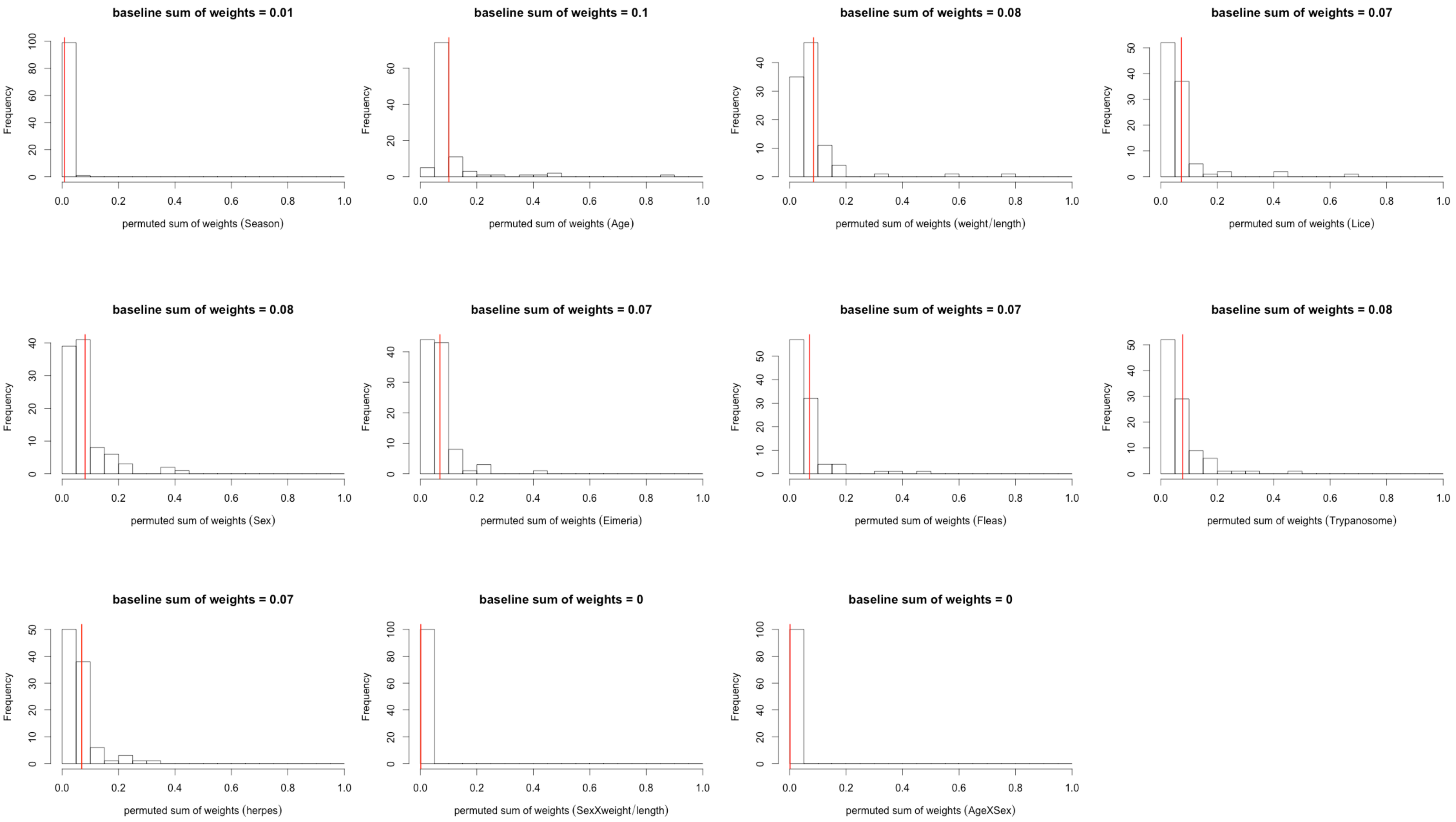

Fig. D Baseline sum of weights for each predictor from 100 permutations of the response variable for LCC and pathogen model. Mean values are indicated with the red line.

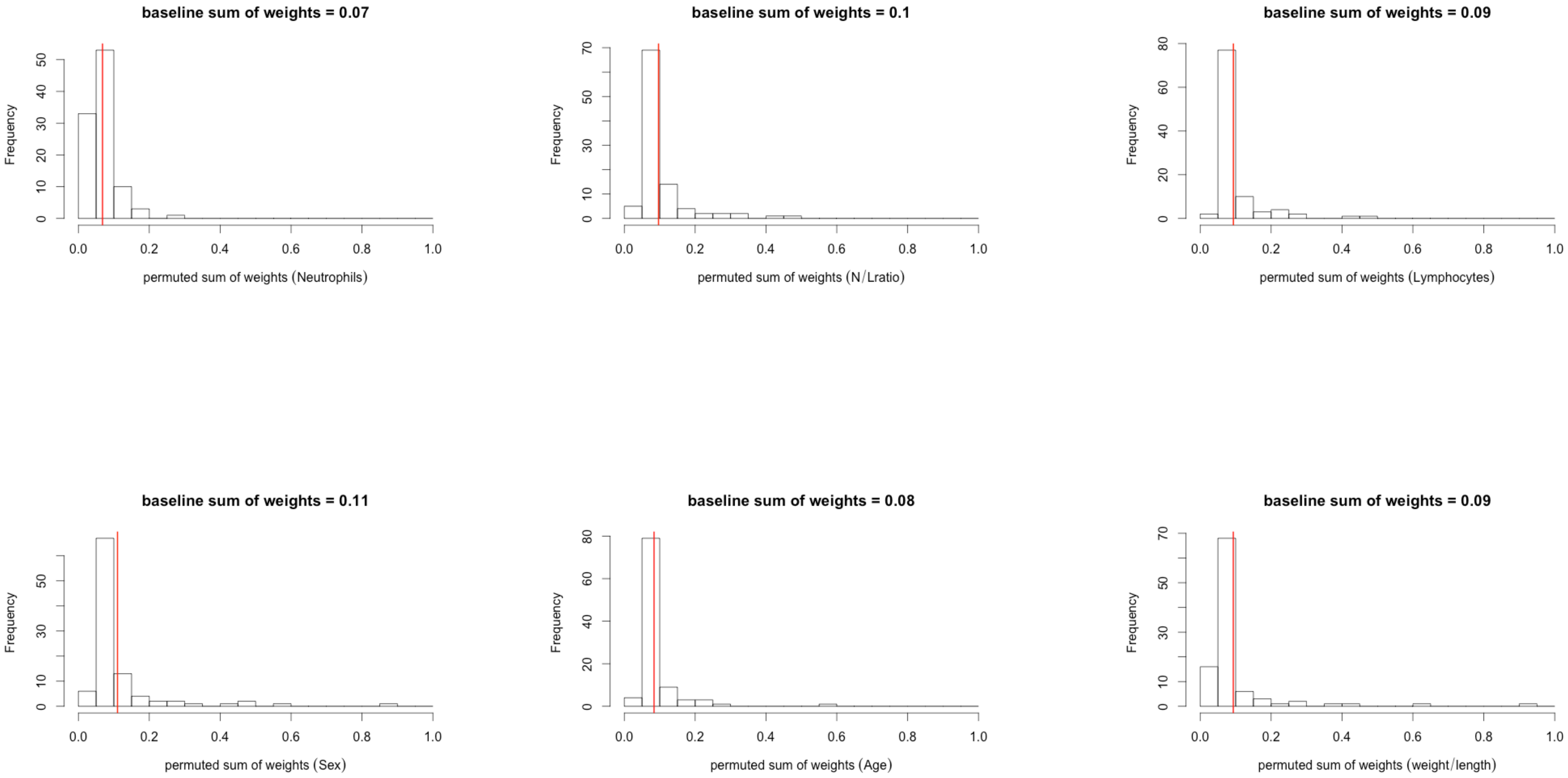

Fig. E Baseline sum of weights for each predictor from 100 permutations of the response variable for LCC and white blood cell counts model. Mean values are indicated with the red line.
